# Oesophageal varices in patients with liver cirrhosis attending a major tertiary hospital in Ghana

**DOI:** 10.11604/pamj.2018.31.230.16657

**Published:** 2018-12-13

**Authors:** Amoako Duah, Kofi Nyaako Nkrumah, Kenneth Tachi

**Affiliations:** 1 St Dominic Hospital, Akwatia, Ghana; 2 Department of Medicine and Therapeutics School of Medicine and Dentistry, College of Health Science, University of Ghana, Korle-Bu, Accra, Ghana

**Keywords:** Prevalence, oesophageal varices, liver cirrhosis, Ghana

## Abstract

**Introduction:**

Oesophageal variceal bleeding is a potentially fatal consequence of portal hypertension in cirrhotic patients. In Ghana, bleeding oesophageal varices (OV) are a significant cause of acute upper gastrointestinal bleeding with comparatively high mortality. This study was to determine the prevalence of OV and its clinical correlate in cirrhotic patients.

**Methods:**

This was a cross sectional hospital based study of 149 subjects with liver cirrhosis from 5^th^November, 2015 to 4^th^ November, 2016. Demographic and other clinical data were collected using standardized questionnaire. Liver function, full blood count, HBsAg and anti-HCV Ab tests were done for all patients. All patients underwent an abdominal ultrasound to assess liver and document ascites. Upper GI endoscopy (UGIE) was done to screen for and grade varices.

**Results:**

A total of 149 patients with a mean age of 45 ± 12.28 years were evaluated. There were 77.85% and 22.15% men and women respectively, with a male to female ratio of 3.5:1. By Child-Pugh Classification, 12 (8.16%) patients were in class A, 64 (43.54%) in class B and 71 (48.3%) in class C at presentation. On UGIE, 135 (90.60%) had varices and 14 patients (9.40%) had no varices. One hundred and eleven of the varices (82.22%) were large varices and the rest (17.78%) small varices.

**Conclusion:**

Majority of cirrhotic patients present late with advance disease to this referral centre. Most have large varices on their first screening endoscopy. Prophylactic treatment should be considered for all cirrhotics especially patients with decompensated liver cirrhosis when UGIE cannot be done immediately.

## Introduction

Liver diseases, especially liver cirrhosis, are common in Ghana and Africa due to the high prevalence of chronic viral hepatitis [1]. Portal hypertension is one of the most significant complications of liver cirrhosis. Gastroesophageal varices are the most important clinical manifestation of portal hypertension. Oesophageal varices (OV) are present at diagnosis in approximately 50% of cirrhotic patients, being more common in Child-Pugh class C patients compared to Child-Pugh class A patients (85% versus 40%) [2, 3]. De novo formation of varices occurs at a rate of 8% per year [3, 4] and the strongest predictor for development of varices in those with cirrhosis who have no varices at the time of initial endoscopic screening is an hepatic venous pressure gradient (HVPG) > 10mmHg [4]. Once varices form, they progress from small to large at a rate of 5-12% per year [5]. Rupture and bleeding of varices portends a poor outcome. Once OV have been identified in a patient with cirrhosis, the risk of variceal bleeding is 25-35% and accounts for 80-90% of bleeding episodes in these patients [5-7]. Bleeding caused by rupture of the OV is associated with a mortality rate of 20% when patients are treated optimally in hospital [8]. The 6 week mortality with each episode of variceal hemorrhage is approximately 15 to 20%, ranging from 0% among patients with Child class A disease to approximately 30% among patients with Child class C disease [9, 10] and up to 70% of untreated patients die within 1 year of the initial bleeding episode [11]. The degree of hepatic decompensation (Child class) is the most important determinant of long term survival after a variceal haemorrhage [12, 13]. Survivors of an episode of active bleeding have a 70% risk of recurrent hemorrhage within one year [14]. The poor outcome of variceal bleeding makes identification of those at high risk and prevention of a bleeding episode critically important [15]. Early diagnosis of OV before the first bleed is essential as studies of primary prophylaxis with nonselective beta blockers have clearly shown that the risk of variceal bleeding can be reduced by 50% to about 15% for large oesophageal varices [16]. In Ghana, bleeding OV are significant cause of acute upper gastrointestinal bleeding with comparatively high mortality [17]. Deaths from OV bleeds, the leading cause of death from acute upper gastro-intestinal haemorrhage rose from 46% in 2010 to 76% in 2013 [17]. Outcomes following acute upper gastro-intestinal bleeding were dismal with some 38% of fatalities occurring within the first 24 hours [17]. There are no published studies describing the prevalence of OV in liver cirrhosis among Ghanaians. This study is therefore to determine the prevalence of OV and its clinical correlates in cirrhotic patients.

## Methods

A formal approval of this study was obtained from the Ethical and Protocol Committee of the University of Ghana School of Medicine and Dentistry. This study was conducted in accordance with the Helsinki Declaration. The research design was a cross -sectional hospital-based study, carried out at the Department of Medicine, Korle-Bu Teaching Hospital (KBTH), Accra, from 5^th^ November, 2015 to 4^th^ November, 2016. All cirrhotic patients who gave their consent and were undergoing their first screening endoscopy for varices were recruited. Their medical records were reviewed to confirm the diagnosis of cirrhosis based on the presence of two or all three of the following [7]; 1. Clinical signs of chronic liver disease (clubbing, palmar erythaema, spider naevi, gynaecomastia, distended abdominal veins, female pubic hair pattern, encephalopathy, splenomegaly or ascites) 2. Impaired liver function test consistent with cirrhosis (elevated INR, and low serum albumin) 3. Ultrasound diagnosis of cirrhosis (Shrunken or enlarged nodular liver with increased echotexture, a blunt edge and distorted architecture, with or without a dilated portal vein, thickened gallbladder wall, splenomegaly or ascites) And/or 4. Diagnosis of cirrhosis based on liver biopsy. Patients with previous diagnosis of OV and on treatment for it were excluded from the study. Those who refuse to consent to the study were also excluded. Patients who met the criteria above were selected using the convenience sampling method After thoroughly explaining the study to patients, those who gave their consent were administered appropriate questionnaire (socio-demographic data and clinical history including alcohol use of the patients were obtained). Alcoholic aetiology was made when patient's declared alcohol consumption was more than 21 units of alcohol for men or 14 units for women per week when measurable or local alcohol beverage consumption was three times per week in the past five years and correlated with biological abnormalities related to alcohol consumption [18]. Ten (10) mls of blood was taken on a single occasion for haematological, biochemical and serological workup. Full blood count and liver chemistry and function test were done. For each patient, a modified Child-Pugh score was calculated [19]. All patients were tested for HBsAg and anti-HCV Ab to determine the cause of liver cirrhosis. Serum antinuclear antibodies (ANA), anti-smooth muscles antibodies (ASMA), serum IgG and anti-liver kidney microsomal (LKM) tests were carried out for patients suspected to have autoimmune hepatitis. Viral aetiology of cirrhosis was considered when one of these serological tests of HBV (HBsAg) or HCV (anti-HCV Ab) was positive. All patients underwent abdominal ultrasound scan after overnight fast and the following details were recorded: maximum vertical span of the liver; nodularity of liver surface; spleen size (Length of its longest axis); and presence of ascites. UGIE was done for assessment of OV. The presence and size of OV were recorded if present. The sizes of the varices were subdivided into two classes-small and large. Small OV defined as varices that flatten with insufflation or minimally protrude into the oesophageal lumen, while large OV defined as varices that protrude into the oesophageal lumen and touch each other (presence of confluence), or that fill at least 50% of the esophageal lumen [20]. Optic and video endoscopes (GIF XQ 10 Olympus) were used. Statistical Package for Social Sciences (SPSS) version 18 data entry template was used for statistical analysis. Descriptive statistics was run for all the variables and data presented in appropriate graphs and tables. Chi square test and T test statistics were used to determine the level of association. A multivariate logistic regression analysis was done for selected binary variables to determine if any of them was a predictor of oesophageal varices. All p-values for this work was two-tailed with p < 0.05 as significant.

## Results

**Figure 1:**
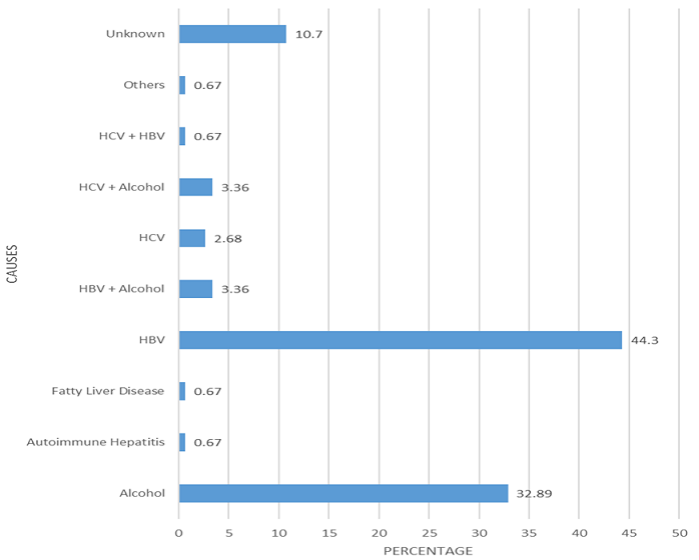
Causes of liver cirrhosis

**Figure 2:**
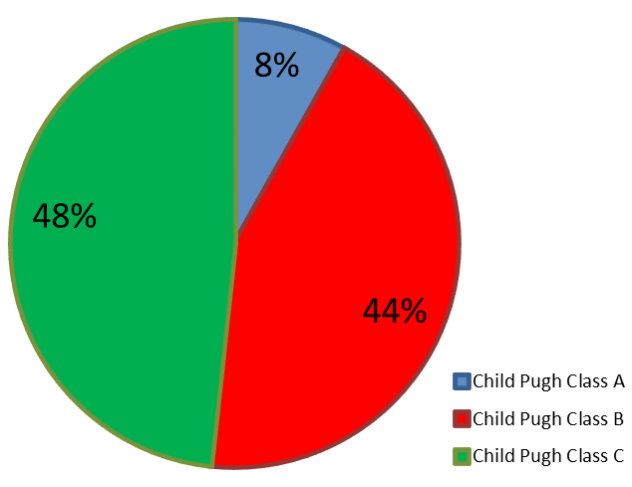
Child pugh classification

A total of 149 cirrhotics were evaluated. This consisted of 33 female and 116 male patients with a male to female ratio of 3.5:1. The mean age of the patients was 45 ± 12.28 years (Table 1). HBV infection was the cause of liver cirrhosis in 44.3% when acting alone and 48.33% when in combination with alcohol and HCV infection. Alcohol alone accounted for 32.89% of causes of liver cirrhosis and 39.61% when combined with HBV and HCV infections. HCV mono-infection, autoimmune hepatitis and fatty liver were uncommon causes Figure 1. Screening upper endoscopy revealed OV in 135 (90.60%) whiles 14 patients (9.40%) had no varices. One hundred and eleven of those with varices (82.22%) were large varices and the rest (17.78%) small varices. Based on Child-Pugh Classification to determine severity of liver disease, 12 (8.16%) patients were classified as class A, 64 (43.54%) as class B and 71 (48.3%) as class C (Figure 2). Clinical features noted to be associated with presence of OV are illustrated in Table 1. Jaundice, haematemesis, melena stools, weight loss and anorexia were found to be significantly associated with presence of OV. However, no significant association was found between pedal oedema, ascites, encephalopathy and the presence of varices. In multivariate analysis none of these clinical features were found to be statistically significant (Table 2). The following laboratory parameters; AST, ALP, GGT were found to be associated with presence of OV among the cirrhotic patients. However none of these were statistically significant on multivariate analysis (Table 2).

**Table 1:** Association of demographic and clinical features of cirrhotic patients with the presence and absence of oesophageal varices

Demographic and clinical characteristics	All (n=149)	Patients With Oesophagel Varices n=135	Patients Without Oesophageal Varices n=14	P-value
Mean age (years)*	45 ± 12.28	135 (90.60%)	14(9.40%)	0.197
**Sex**				
Male	116	106 (91.38)	14 (8.62%)	0.840
Female	33	29 (87.88%)	5(12.12%)	
**Clinical findings [n (%)]**				
Ascites	122 (81.88)	109 (89.34)	13 (10.66)	0.263
Weight Loss	59 (39.60)	50 (84.75)	9 (15.25)	0.047†
Pedal oedema	60 (40.27)	55 (91.67)	5 (8.33)	0.715
Mealena Stools	74 (49.66)	73 (98.65)	1 (1.35)	0.001†
Haematemesis	85 (57.05)	83 (97.65)	2 (2.35)	0.001†
Hepatic encephalopathy	28 (18.79)	26 (92.86)	2 (7.14)	0.650
Jaundice	59 (39.60)	50 (84.75)	9 (15.25)	0.047†
Anorexia	35 (23.49)	27 (77.14)	8 (22.86)	0.002†
Child Pugh Score*	9.57 ± 2.26	9.53 ± 2.30	10.0 ± 1.88	0.458
Child Pugh Class A	12 (8.16)	12 (100.0)	0.0 (0)	
Child Pugh Class B	64 (43.54)	57 (89.06)	0 (0.0)	0.491
Child Pugh Class C	71 (48.3)	64 (90.14)	64 (90.14)	
Systolic BP*	112.70 ± 15.67	112.63 ± 16.09	113.29 ± 11.36	0.883
Diastolic BP*	68.44 ± 12.82	68.46 ± 13.13	68.21 ± 9.74	0.945
BMI*	24.35 ± 3.40	24.41 ± 3.50	23.87 ± 2.25	0.577

Data are presented as frequencies and percentages. Categorical data were analyzed using Chi-squared and continuous data were analyzed using T-test statistics all at 95% confidence intervals.

* Data expressed as Mean ± SD

**Table 2:** Multiple logistic regression model of Independent risk factors of oesophageal varices (dependent variable (present/absent))

Independent Risk factors	Adjusted Odds Ratio (OR)	Standard error	95% CI	p-value
Jaundice	1.58	1.28	0.32 - 7.75	0.57
Haematemesis	1.31	1.46	0.15 - 11.53	0.81
Mealena Stools	38.95	82.15	0.62 - 2431.00	0.08
Weight Loss	0.77	0.69	0.13 - 4.491	0.78
Anorexia	0.20	0.18	0.04 - 1.13	0.07
AST	1.00	0.00	0.99 - 1.01	0.31
ALP	1.00	0.00	0.99 - 1.00	0.27
GGT	1.00	0.00	0.99 - 1.01	0.10

LR Chi2: Likelihood Ratio Chi-Squared. P<0.05 was considered to be significant

## Discussion

The mean age of the respondents was 45 ± 12.28 years. This is worrying but not surprising; worrying because this age bracket constitutes the working population who unfortunately are bearing the brunt of liver cirrhosis with implications on productivity and burden on the society. As expected however, this is the period when complications of common afflictions of the liver begin to occur. Similar age cohorts have been reported from similar studies in this country [21] and other Africa countries in patients with liver cirrhosis [18, 22]. However, the mean age in other studies done in western countries were higher than this study [23, 24]. Variations in the mean age in different geographic regions are likely to be related to differences in the aetiologies and especially prevalence of hepatitis viruses in the populations, as well as the timing of the spread of the viral infection and the age of individuals at the time of the infection. Chronic hepatitis B was either alone or in combination with other risk factors was identified as the leading cause of cirrhosis whilst HCV was a less important (6.71%) cause. This is consistent with the high prevalence of HBV (13%) and relatively low prevalence of HCV(3.0%) in Ghana [25, 26]. This finding is in parallel with reports in the literature from West Africa and other hepatitis B endemic countries [21, 27]. Alcohol abuse was the second common cause of chronic liver disease in this study which implies that alcohol is a significant cause of liver cirrhosis in patients attending clinic at KBTH. This is a public health concern; therefore society should be educated on the harmful effect of alcohol abuse on the liver. A study by Abel *et al*. in Ethiopia [28], found HBV and HCV as the major causes of liver cirrhosis whiles in Sudan [29] alcohol abuse and HBV were the common causes. However in Brazil [30] alcohol was the commonest cause and in United States of America [31] HCV and alcohol were the major causes. The causes of liver cirrhosis are the same globally but the percentage of individual causes varies from one country to another country. NAFLD was not an important cause of cirrhosis in this study although, globally, it is increasingly becoming an important aetiological factor. The reasons for this difference may be due to increasing rate of obesity in the western countries and Asia compared to African countries [32].

Global estimates of prevalence of OV is approximately 50% of cirrhotic patients, being more common in Child-Pugh class C patients compared to Child-Pugh class A patients (85% versus 40%) [2, 3]. (This study showed a high rate of 90.6% OV among cirrhotic patients screened at the KBTH. The high prevalence rate of this study is similar to 83% reported by Lay *et al.* [33]. The large number of decompensated liver cirrhosis patients involved in this study may partially explain the high prevalence of varices in this study. Also this study did not exclude patients who presented with upper gastrointestinal bleeding. The majority, 82.22% of those with varices had large OV and most of the patients in this study were in Child-Pugh class B and C. Even though this study did not assess the duration of illness, it is likely that late presentation is a factor for the many large varices noted. Other possible causes of delay may be from delay by clinicians in requesting the upper GI endoscopy to screen for varices or delay in accessing endoscopy service, often due to financial difficulties and unavailability/breakdown of the facilities. The high prevalence of large OV (82.22%) is similar to 70% reported in Nigeria [34] and 70.2% in Côte d'Ivoire [18]. Late presentation and the large number of subjects with advanced liver cirrhosis accounted for this similarity. Knowing that large varices are at highest risk of bleeding and a first variceal bleed carries 30% mortality, it means that many of our cirrhotics are sitting on time bombs. None of the clinical features and laboratory parameters were significantly associated with the occurrence of varices in this study. This is in contrast with studies conducted by Chalasani *et al*. [35] who found platelet count and splenomegaly as independent predictors of varices. Achinge *et al.* [34] also found age, shrunken liver span and low platelet count as predictors of varices. The reasons for these variations are unclear. This study is not without limitations. The diagnosis of liver cirrhosis in this study was based mainly on clinical, laboratory and radiologic examinations. This method of diagnosis without any histologic confirmation may be less accurate as other causes of portal hypertension leading to OV could have been included. Another limitation of the study was the fact that it was a hospital based study and only patients with distressing symptoms would more likely be recruited hence the association of varices with advanced disease in newly diagnosed cirrhotic patients. A community based study would have eliminated this error but this would be expensive and time consuming and probably impracticable.

## Conclusion

Most patients with cirrhosis at the KBTH are middle aged men and chronic HBV infection is the leading aetiological factor underlying cirrhosis. Alcohol use was the second commonest cause of liver cirrhosis but HCV and NAFLD are not major causes of cirrhosis at KBTH. Presentation for screening upper endoscopy is often late and with severe liver disease. Majority of cirrhotics have varices at endoscopy and with most being large varices. Prophylactic treatment should be considered for all cirrhotics when upper GI endoscopy cannot be done immediately. Efforts at controlling HBV including universal vaccination at birth and access to testing and treatment should be intensified. Religious and Health education to prohibit excessive ethanol intake should be encouraged. A similar study should be done on a larger scale country-wide in multiple centres and regions to get a well-balanced prevalence of OV.

### What is known about this topic

- Point prevalence of medium/large varices is approximately 15-25%, the majority of subjects undergoing screening endoscopy either do not have varices or have varices that do not require prophylactic therapy;
- The use of simple non-invasive markers, such as increased INR, low serum albumin, a low platelet count, Chug-Pugh classification may help physicians to predict which patients are likely to have large varices.

### What this study adds

- A large proportion of Ghanaians with liver cirrhosis attending Korle-Bu teaching hospital have large oesophageal varices at diagnosis and at their first endoscopic screening;
- None of the laboratory parameters, such as increased INR, low serum albumin, low platelet, Chug-Pugh classification were significantly associated with the occurrence of varices in this study.

## Competing interests

The authors declare no competing interests.
